# Modelling Macular Edema: The Effect of IL-6 and IL-6R Blockade on Human Blood–Retinal Barrier Integrity In Vitro

**DOI:** 10.1167/tvst.8.5.32

**Published:** 2019-10-28

**Authors:** Marina Mesquida, Faye Drawnel, Philippa J. Lait, David A. Copland, Madeleine L. Stimpson, Victor Llorenç, Maite Sainz de la Maza, Alfredo Adan, Gabriella Widmer, Pamela Strassburger, Sascha Fauser, Andrew D. Dick, Richard W. J. Lee, Blanca Molins

**Affiliations:** 1 Institut d'Investigacions Biomediques August Pi i Sunyer (IDIBAPS) and Hospital Clínic de Barcelona, Spain; 2 Roche Pharma Research and Early Development, Roche Innocation Centre Basel, Switzerland; 3 Academic Unit of Ophthalmology, Translational Health Sciences, University of Bristol, Bristol, UK; 4 National Institute for Health Research (NIHR) Biomedical Research Centre at Moorfields Eye Hospital and University College London Institute of Ophthalmology, London, UK

**Keywords:** interleukin-6, macular edema, blood retinal barrier, inflammation

## Abstract

**Purpose:**

Macular edema (ME) is a leading cause of visual loss in a range of retinal diseases and despite the use of antivascular endothelial growth factor (anti-VEGF) agents, its successful treatment remains a major clinical challenge. Based on the indirect clinical evidence that interleukin-6 (IL-6) is a key additional candidate mediator of ME, we interrogated the effect of IL-6 on blood–retinal barrier (BRB) integrity in vitro.

**Methods:**

Human retinal pigment epithelial cell (ARPE-19) and human retinal microvascular endothelial cell (HRMEC) monolayers were used to mimic the outer and inner BRB, respectively. Their paracellular permeability was assessed by measuring the passive permeation of 40 kDa fluorescein isothiocyanate (FITC)-dextran across confluent cells in the presence of IL-6. Transendothelial/epithelial electrical resistance (TEER) then was measured and the distribution of the tight junction protein ZO-1 was assessed by immunofluorescence using confocal microscopy.

**Results:**

Treatment with IL-6 for 48 hours significantly increased the diffusion rate of FITC-dextran, decreased TEER, and disrupted the distribution of ZO-1 in ARPE-19 cells, which constitutively express the IL-6 transmembrane receptor, and this was reversed with IL-6R blockade. In contrast, IL-6 did not affect the paracellular permeability, TEER, or ZO-1 distribution in HRMECs.

**Conclusions:**

These in vitro data support the hypothesis that IL-6 reversibly disrupts the integrity of ARPE-19 cells, but it does not affect HRMECs.

**Translational Relevance:**

IL-6 is a candidate therapeutic target in the treatment of outer BRB driven ME.

## Introduction

Macular edema (ME) is a leading cause of visual impairment, and is associated with retinal barrier dysfunction and the consequent accumulation of extracellular fluid within the central retina.^1^,^2^ Although a comprehensive understanding of its underlying pathophysiology remains elusive, vascular endothelial growth factor (VEGF) is known to have a key role in ME associated with retinal ischemia and inflammation, and intraocular injections of anti-VEGF agents are now standard treatment for diabetic macula edema (DME) and retinal vein occlusion (RVO), with ranibizumab (Lucentis; Genentech, South San Francisco, CA) and aflibercept (Eylea; Regeneron Pharmaceuticals, Tarrytown, NY) being approved by the United States Food and Drug Administration and European Medicines Agency for these indications. Furthermore, bevacizumab (Avastin; Roche, Basel, Switzerland) also is used off-label for treating retinal diseases, including the orphan indication of uveitic ME (UME).^3^–^5^ However, despite the visual benefits anti-VEGF treatments have achieved, they fail to resolve ME in >20% of patients with DME^6^ and in >40% of patients with RVO and uveitis/UME,^7^,^8^ suggesting that there also are important alternative mediators of retinal barrier dysfunction. Principal among these is the cytokine interleukin-6 (IL-6) as its intraocular concentration correlates with the severity of ME in a diverse range of retinal pathologies, including diabetic retinopathy, DME, RVO, and uveitis.^9^,^10^–^12^ The impact of IL-6 on vascular permeability already has been studied in a variety of nonocular tissues and cancer.^13^,^14^ Our group and others have reported previously that in patients with refractory UME, systemic inhibition of IL-6 signaling with the IL-6 receptor (IL-6R) monoclonal antibody tocilizumab (TCZ – Actemra; Hoffmann-La Roche Ltd, Basel, Switzerland) was beneficial at 6, 12, and 24 months,^15^–^17^ in particular with regard to achieving a reduction in central retinal thickness where other therapies had failed. However, the direct effect of IL-6 on the human blood retinal barrier (BRB) is yet to be fully elucidated. Therefore, we designed the present laboratory study to model the inner BRB using human retinal microvascular endothelial cells (HRMEC) and the outer BRB using human retinal pigment epithelial (ARPE-19) cells, with the goal of interrogating the effects of IL-6 and IL-6R blockade on in vitro measures of BRB integrity.

## Materials and Methods

### Cell Culture

To mimic the outer BRB, a spontaneously arising ARPE-19 cell line was obtained from the American Type Culture Collection (ATCC). ARPE-19 cells then were cultured in a 50:50 mixture of Dulbecco modified Eagle medium (DMEM) and Ham's F12 (PAA) supplemented with 10% fetal bovine serum (FBS, PAA), 2 mM L-glutamine (PAA), 100 U/mL penicillin (PAA), 0.1 mg/mL streptomycin (PAA), and 1 mM sodium pyruvate (Sigma-Aldrich Corp., St. Louis, MO) in a humidified incubator at 37°C in 5% CO_2_. Cells were passaged every 4 to 5 days by trypsinization.

For the inner BRB, HRMEC cells (ACBRI 181) purchased from Cell Systems Corporation (Kirkland, WA) were cultured in MV2 growth medium (PromoCell) containing 5.5 mM glucose, 0.5 ng/mL VEGF, 5% FBS, 5 ng/mL EGF, 10 ng/mL bFGF, 20 ng/mL IGF, 1 μg/mL ascorbic acid, 100 U/mL penicillin, and 0.2 μg/mL hydrocortisone. Because VEGF is a major initiator of barrier breakdown we tested the effect of VEGF-A on transendothelial electrical resistance (TEER) in HRMEC with concentrations ranging from 0.3 to 80 ng/mL (Supplementary Fig. S1), observing that at concentrations below 5 ng/mL, VEGF-A does not induce significant loss of endothelial cell barrier. This confirmed that the concentrations of VEGF-A in the MV2 medium used for our experiments is 10-fold below the threshold needed to affect HRMEC integrity in our in vitro system, and, hence, this will not influence our results. Cells were fed triweekly and passaged every 4 to 5 days by trypsinization. For all experiments, cells were used after 4 to 9 passages. For experiments measuring IL-6R expression in HRMEC, cells were cultured in either full growth medium as above or in starvation medium, composed of MV2 medium (PromoCell) containing 5.5 mM glucose and 0.5% FBS only.

For immunofluorescence, cell suspensions of 1 × 10^5^ cells/mL were seeded onto glass coverslips and incubated overnight. Cells then were stimulated with or without IL-6 (R & D Systems, Minneapolis, MN) for 24 to 48 hours. To test the ability of IL-6R blockade to reverse the effects IL-6, cells were treated with 200 ng/mL TCZ either simultaneously or 24 hours after IL-6 stimulation.

### Permeability Assay

The paracellular permeability of ARPE-19 and HRMEC monolayers was assessed by measuring the passive permeation of fluorescein isothiocyanate (FITC)-dextran (40 kDa, Sigma-Aldrich Corp.) across confluent cells grown on Transwell filters (Costar, 12 mm diameter, 0.4 μm pore size). For monolayer culture, cells were seeded at 2 × 10^5^ cells/cm^2^.

For ARPE-19 cells, monolayers were cultured for 2 to 3 weeks in medium containing 1% FBS, changing the medium every 3 days. At day 19, cells were treated with IL-6 (200 and 400 ng/mL) for 48 hours. At day 21, 500 μg/mL fluorescein isothiocyanate FITC-dextran were added to the apical compartment of the chamber and samples (200 μL) from the basal medium (lower chamber) were collected 120 minutes after addition of FITC-dextran. The absorbance of basal and apical medium samples was measured at 485 nm of excitation and 528 nm of emission in a microplate reader (SpectraMax Gemini; Molecular Devices, San Jose, CA). Each condition was assayed in triplicate and repeated in at least three independent experiments. The diffusion rate was expressed as a percentage and calculated as follows: (amount of dextran lower chamber) × 100/(amount of dextran upper chamber).

For HRMEC permeability assays, cells were cultured on Transwell filters for 14 days. At day 12, cells were treated with IL-6 (200 and 400 ng/mL) for 48 hours and at day 14, FITC-dextran was added to the apical compartment of the chamber and the paracellular permeability was measured as described above.

### Measurement of TEER

For ARPE-19 cells (grown in monolayers on Transwell filters as described above), TEER was measured using a commercial electrical resistance system (Millicell; Millipore). TEER values were calculated by subtracting the value of a blank (Transwell filter without cells) and expressed as resistance/area (Ω/cm^2^) relative to the resistance/area of the baseline at day 3. Measurements were repeated at least three times for each filter, and each experiment was repeated at least four times using two filters.

In HRMEC, TEER was measured using the CellZscope system (NanoAnalytics, Munster, Germany) in cell monolayers grown on transwells as described above. TEER was measured continuously in a CO_2_ incubator at 37°C. TEER values were expressed as resistance/area (Ω/cm^2^) relative to the resistance before the addition of IL-6. Measurements were collected from three filters per experimental condition.

### Immunofluorescence

The distribution of the tight junction protein 1, also known as ZO-1, in ARPE-19 monolayers was examined by immunofluorescence. Cells grown on cover slides were fixed with 4% paraformaldehyde in PBS for 15 minutes at room temperature (RT) and washed three times with PBS. Cells then were permeabilized with 0.2% Triton X-100 in PBS for 15 minutes and blocked twice with filtered 1% bovine serum albumin. Cells were incubated with primary antibody anti-ZO-1 (Invitrogen, Carlsbad, CA) overnight, before washing with PBS, and final incubations with secondary antibody Alexa Fluor anti-mouse 488 IgG for 1 hour at RT. Nuclei were counterstained with 4′,6-diamidino-2-phenylendole (DAPI). Controls were stained with secondary antibodies only. Stained cells were washed and covered with Prolong Gold antifade reagent (Life Technologies, Carlsbad, CA). Images of immunostained cells were captured using a Leica TCS SP5 (Leica, Wetzlar, Germany) confocal laser scanning microscope, imaged with an APO ×63 objective. The area and intensity covered by membrane ZO-1 were calculated using Image J.

### Quantification of IL-6R in HRMEC

To quantify HRMEC IL-6R expression, cells were plated at 30,000 cells per well in 96-well culture plates and grown in either growth or starvation medium for 72 hours. At the end of the growth period, conditioned medium was harvested and cell cultures washed twice with PBS-Ca^2+^, −Mg^2+^ (Life technologies), and once with 50 μL dissociation reagent (Life Technologies). After washing, cells were detached from the plates by incubation with 100 μL cell dissociation reagent and transferred to a flow cytometry plate (Corning, Corning, NY). The original wells were washed once with 100 μL PBS-Ca^2+^, −Mg^2+^ (Life Technologies) and the wash solution containing remaining cells added to the flow cytometry plate. Cells were pelleted by centrifugation at 300*g* for 5 minutes and the supernatant discarded. Pellets were resuspended in MACS buffer (PBS containing 2% FBS and 2 mM ethylenediaminetetraacetic acid [EDTA]) containing 10 μg/mL human IgG (Sigma-Aldrich Corp.) to block nonspecific binding sites and incubated for 15 minutes at RT. Following blocking of nonspecific binding, anti-human IL-6Ra-PE, anti-human VEGF-R1-PE or isotype control antibody (R & D Systems) were added to the cells and the reaction incubated for 45 minutes at 2°C to 8°C. Following staining, cells were pelleted by centrifugation at 300*g* for 5 minutes and the pellet resuspended in 110 μL MACS buffer. PE fluorescence was measured using a BD LSRII flow cytometer.

Soluble IL-6R was quantified in conditioned growth and starvation medium using a Luminex high-performance IL-6R assay (R & D Systems), according to the manufacturer's instructions. Nonconditioned medium also was quantified as a control. Samples were diluted 1:1 and measured in duplicate using a Luminex 200 system.

### Statistical Analysis

Results are expressed as mean ± standard deviation (SD). Student's *t*-test or analysis of variance (ANOVA) as appropriate were used to determine statistical significance between treatments. *P* < 0.05 was considered significant. All calculations were performed using GraphPad Prism (GraphPad Software, San Diego, CA).

## Results

### IL-6 Increases Paracellular Permeability With Concomitant Decrease in TEER in ARPE-19 Monolayers

**Figure 1:**
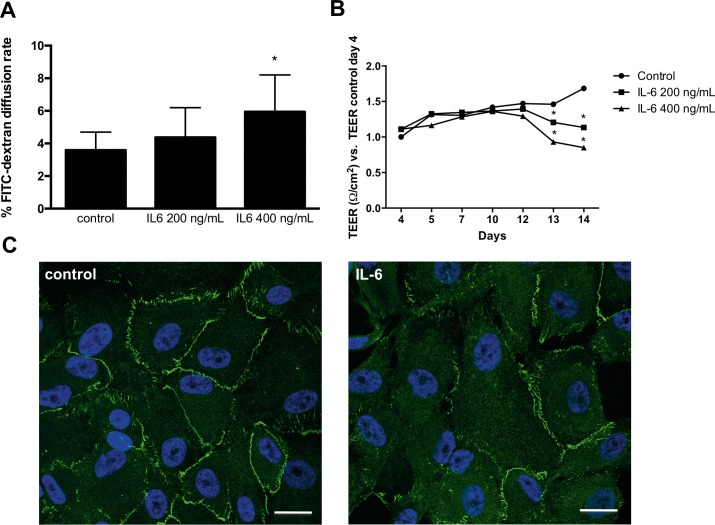
Effect of IL-6 on paracellular permeability, TEER, and ZO-1 distribution in ARPE-19 cells. ARPE-19 cells grown on Transwells were treated with different concentrations of IL-6 for 48 hours and the diffusion rate of FITC-dextran (N = 7) (A) and TEER (B) were determined (N = 4). Values are expressed as mean ± SD and statistical analysis was performed by Student's t-test. *P < 0.05. ARPE-19 cells also were treated with IL-6 (400 ng/mL) for 48 hours, fixed, and immunostained with anti ZO-1 (green) and DAPI (blue) (C). Images shown are representative of three independent experiments. Scale bar: 25 μm.

To determine the effect of IL-6 on outer BRB integrity, we first assessed the effect of IL-6 on the paracellular permeability of ARPE-19 cell monolayers, which constitutively express the IL-6 transmembrane receptor,^18^,^19^ by measuring the transcellular diffusion rate of FITC-dextran (40 kDa). As shown in Figure 1A, treatment with IL-6 (200 and 400 ng/mL) for 48 hours significantly increased the diffusion rate of FITC-dextran in a dose-dependent manner (*P* < 0.05 vs. control). We next determined the effect of IL-6 on ARPE-19 monolayer TEER. In line with our findings on paracellular permeability, TEER was significantly reduced as shown in Figure 1B, and this was evident after 24 hours of stimulation (day 13).

### IL-6 Alters the Expression of ZO-1 in ARPE-19 Cells

As we had demonstrated increased permeability and decreased TEER in ARPE-19 monolayers we proceeded to test the effect of IL-6 on tight junctions by assessing the distribution of ZO-1. In untreated cells, ZO-1 expression was continuous and regular. The expression demarcated the cell membranes and was particularly intense at contact points in the monolayer. However, following exposure to 400 ng/mL of IL-6 (the concentration that induced the greatest effect in permeability and TEER disruption) for 48 hours, the monolayer distribution of ZO-1 was disturbed. The abnormal distribution of ZO-1 manifested as diffuse cytoplasmic distribution and fragmented membrane staining (Figs. 1C, 1D).

### IL-6R Blockade Inhibits IL-6-Induced Outer BRB Disruption

**Figure 2:**
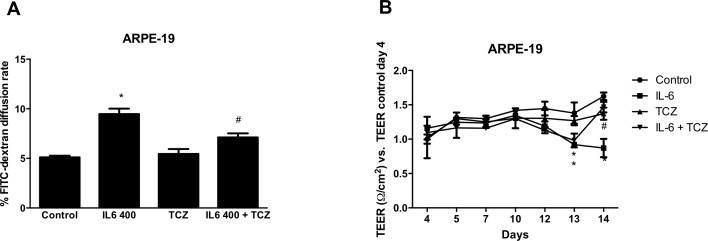
Effect of TCZ on IL-6–induced barrier disruption. ARPE-19 cells grown on filters were treated with IL-6 for 24 hours and then with TCZ for 24 hours. Cell permeability as determined by FITC-diffusion rate (A) and TEER (B) were measured (N = 5). Values are expressed as mean ± SD and statistical analysis was performed by Student's t-test. *P < 0.05 vs. control, #P < 0.05 versus IL-6 + TCZ.

To assess whether IL-6R inhibition reversed IL-6–induced barrier disruption, ARPE-19 cells grown on filters to confluence were treated with TCZ (400 ng/mL) for 24 hours following IL-6 stimulation. The paracellular permeability and TEER was determined after a further 24 hours. As shown in Figure 2, TCZ significantly reversed IL-6–induced barrier disruption. These data demonstrated a significant TCZ associated reduction in (**A**) paracellular permeability and **(B)** TEER in ARPE-19 monolayers (*P* < 0.05).

**Figure 3:**
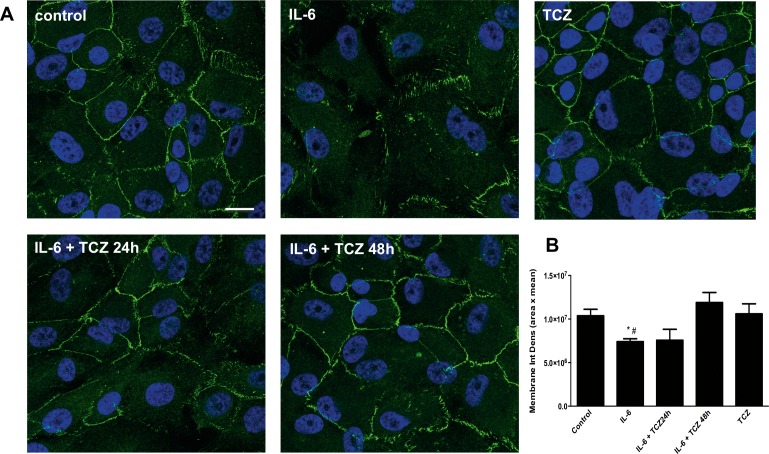
Effect of IL-6 blockade on the distribution of ZO-1 in ARPE-19 cells. Cells were treated with IL-6 (400 ng/mL) for 48 hours, and TCZ was either added simultaneously with IL-6 or 24 hours afterwards. Cells then were fixed and immunostained with anti ZO-1 (green) and DAPI (blue). Images shown are representative of three independent experiments (A). Scale bar: 25 μm. Membrane ZO-1 staining was quantified with Image J measuring the Integrated density (area times mean fluorescence intensity (B). Values are expressed as mean ± SD and statistical analysis was performed by Student's t-test. *P < 0.05 versus control, #P < 0.05 versus IL-6 + TCZ (N = 5).

Finally, to test the ability of IL-6R inhibition to prevent IL-6–induced barrier disruption, ZO-1 distribution also was determined in ARPE-19 cells (Fig. 3) treated with TCZ. In these experiments, TCZ was either added simultaneously with IL-6 for 48 or 24 hours after IL-6 stimulation. TCZ was able to restore ZO-1 distribution in IL-6–treated cells when added simultaneously with IL-6. Conversely, when cells were treated with TCZ alone in a deferred manner, ZO-1 distribution was similar to that of untreated cells.

### IL-6 Fails to Induce Disruption of the Inner BRB

**Figure 4:**
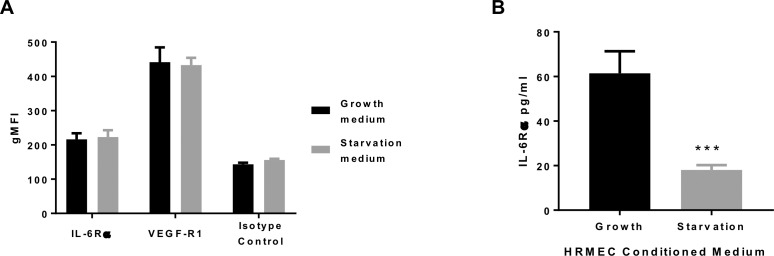
IL-6R expression in HRMEC. (A) IL-6R and VEGF-R1 surface expression in HRMEC grown in full growth or starvation medium were quantified using flow cytometry. Mean fluorescence intensity (MFI) of the labeling produced by the specific antibody is shown in comparison with the labeling produced by the isotype control antibody. Values represent mean ± SD, n = 4 per condition. (B) Soluble IL-6R detection in conditioned medium from HRMEC grown under full growth or starvation conditions. Values are expressed as mean ± SD, n = 4 per condition. Soluble IL-6R was undetectable in nonconditioned medium.

IL-6 can activate cellular signaling via two mechanisms: the classic or *cis* form of signaling, triggered by the binding of the ligand to transmembrane IL-6R, and the trans-signaling pathway induced by activity of the soluble IL-6R on ubiquitously-expressed gp130.^18^ To determine whether IL-6–induced endothelial barrier breakdown could be triggered by IL-6–signaling as observed in ARPE-19 cells, we investigated IL-6R expression in HRMEC. Using flow cytometry, we did not observe expression of transmembrane IL-6R in HRMEC cultured in either full growth or starvation medium, when compared to the labeling produced by the isotype control antibody (Fig. 4A). As expected, surface expression of the endothelial cell marker VEGF-R1 was readily detectable (Fig. 4A).

**Figure 5:**
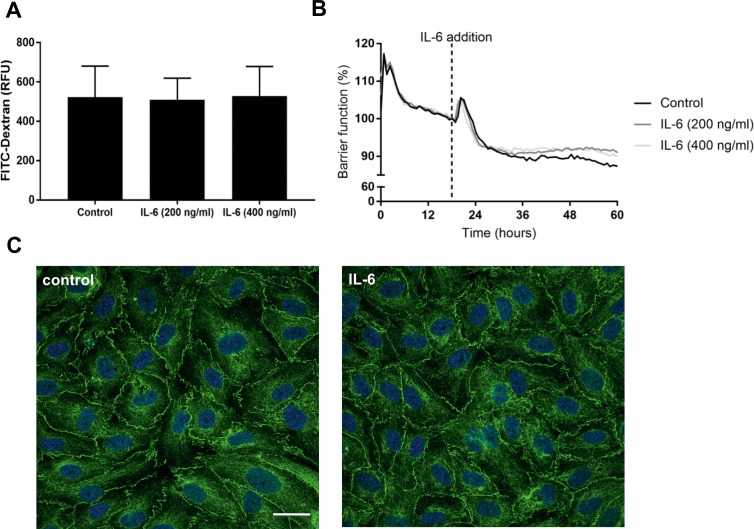
Effect of IL-6 on paracellular permeability, TEER, and distribution of ZO-1 in HRMECs. HRMECs grown on Transwells were treated with different concentrations of IL-6 for 48 hours and the diffusion rate of FITC-dextran (n = 12) (A) at 90 minutes and TEER (n = 3) (B) were determined. Values are expressed as mean ± SD. For immunofluorescence, cells were treated with IL-6 (400 ng/mL) for 48 hours, then fixed and immunostained with anti ZO-1 (green) and DAPI (blue). Images shown are representative of three independent experiments (C). Scale bar: 25 μm. ZO-1 staining was quantified with Image J measuring the ratio of membrane and cytoplasm Integrated density (area times mean fluorescence intensity). No significant differences were observed in any of these measures of barrier integrity.

Consequently, we measured sIL-6R within the conditioned medium of HRMEC cultured under growth or starvation conditions. We found that sIL-6R was detectable at a low level in HRMEC conditioned medium and significantly enriched under full growth conditions. Contrarily, IL-6R was undetectable in nonconditioned HRMEC medium (Fig. 4B). However, this enrichment of sIL-6R in full growth conditions remained insufficient to mediate IL-6–induced barrier breakdown as the transcellular diffusion rate of FITC-dextran, TEER and ZO-1 distribution was unaltered by the addition of IL-6 alone to our HRMEC cultures (Fig. 5). The endogenous secretion of IL-6 by HRMEC was very low (Supplementary Fig. S2).

## Discussion

The functional integrity of the inner and outer BRB is critical for the maintenance of neural retinal health and is dependent on the expression of transmembrane proteins, which are anchored to the actin cytoskeleton.^20^ This permits restricted paracellular permeability and high TEER.^21^,^22^ Our results suggested that the effects of IL-6 compromise this barrier function in retinal microvascular endothelial and retinal pigment epithelial cells. Furthermore, changes in the distribution of ZO-1 are likely to have altered the diffusion rate of FITC-dextran in ARPE-19 cells as IL-6 clearly decreased ZO-1 membrane staining. However, tight junctions are highly complex structures, and the relative contribution of the various proteins to the barrier function remains unclear. Thus, in addition to the disorganization of ZO-1, IL-6 also might modulate other pathways associated with cellular permeability.^1^

Our findings are consistent with prior reports implicating IL-6 in the pathogenesis of ME in human and preclinical in vitro and in vivo experimental systems,^22^–^26^ by decreasing expression of VE-cadherin, occludin, and claudin-5,^27^ and increasing VEGF-mediated vascular permeability via STAT3 activation.^28^,^29^ However, our human-derived cell cultures imply that in man, the principal effect of IL-6 is on retinal pigment epithelial cells, that is, the outer BRB. In our HRMEC assay system, we did not detect expression of cell-surface transmembrane IL-6R, but low levels of soluble IL-6R were quantifiable in the conditioned medium of these cells and enriched under conditions of cell growth. We suggested that this soluble IL-6R is either produced by the cleavage of a limited quantity of transmembrane IL-6R not easily detectable by flow cytometry or directly by the production and release of alternatively-spliced soluble IL-6R.^30^,^31^ Nonetheless, this was in insufficient concentrations to mediate loss of HRMEC integrity in our in vitro system. However, in vivo, soluble IL-6R has been detected in the aqueous humor of eyes with uveitis,^32^ and it is plausible that if this is present in higher concentrations than detected in our cultures, inner BRB disruption still may ensue. Hence, further investigation of the source of soluble IL-6R in these cellular systems, as well as in vivo, is warranted to fully interrogate whether IL-6 trans-signaling may still contribute to breakdown of the inner BRB and consequent ME.

In keeping with this, the main limitation of our study was the use of monoculture systems to mimic the inner and outer BRB. Although HRMECs are isolated from normal human retinal tissue, the human ARPE-19 cell line has been criticized for the divergence in some of its characteristics from normal healthy human RPE.^33^ However, these limitations were partially addressed by the use of Transwell inserts instead of traditional cell culture plates as these allow ARPE-19 polarization. Future studies would nonetheless benefit from the use of pluripotent stem cell-derived RPE and organ-on-a-chip approaches involving multiple cell types to better characterize the BRB. Another caveat of our work is the use of high concentrations of IL-6. The range of IL-6 concentrations previously reported in the context of in vitro systems is highly variable with some investigators matching the concentrations applied in our experiments,^34^–^36^ while others use concentrations in the range of 20 to 100 ng/mL.^28^,^29^ This lack of standardization threatens comparability between studies, and it is possible that the concentrations used in our in vitro systems will have limited relevance in vivo.

In summary, our data supported a key role for IL-6 in disrupting the outer BRB as its addition to in vitro cultures of ARPE-19 cells increased paracellular permeability, decreased TEER, and altered ZO-1 distribution, and these effects were abrogated by blockade of the IL-6R with TCZ. However, IL-6–induced disruption of HRMEC integrity was not seen at the concentrations of soluble IL-6R present in our in vitro culture system. Nonetheless, our observed effect of IL-6 on the integrity of the outer BRB support the continued evaluation of the inhibition of combinations of soluble mediators of vascular permeability, including IL-6 using enhanced in vitro and in vivo experimental systems with a view to clinical translation for sight-threatening ME associated with diabetic retinopathy, RVO, and uveitis.

## Supplementary Material

Supplement 1
