# Supplementary material for: Modelling Macular Edema: The Effect of IL-6 and IL-6R Blockade on Human Blood–Retinal Barrier Integrity In Vitro
Source: Transl Vis Sci Technol. 2019 Oct 28;8(5):32. doi: 10.1167/tvst.8.5.32 (PMC6819001; doi:10.1167/tvst.8.5.32)
Supplement: Supplement 1 [file tvst-08-05-18_s01.pdf]

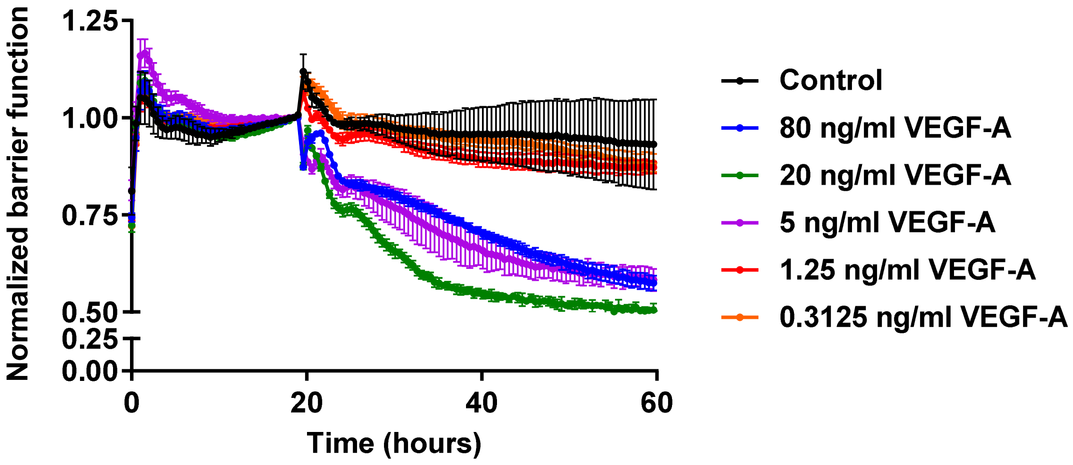

Supplementary Figure S1

Supplementary Figure S1: Dose-response effect of VEGF-A on TEER in HRMEC. HRMECs were cultured in starvation medium (MV2 medium + 0.5% FPS, but lacking all other growth factors) with different concentrations of VEGF-A. TEER was continuously monitored for up to 60 hours. Because full MV2 media contains 0.5 ng/mL of VEGF-A, we tested concentrations in the range of 0.3 to 80 ng/mL, observing that at concentrations below 5ng/mL, VEGF-A does not induce significant loss of endothelial cell barrier. This confirmed that the concentrations of VEGF-A in the MV2 medium used for our experiments is ten-fold below the threshold needed to affect HRMEC integrity in our *in vitro* system.

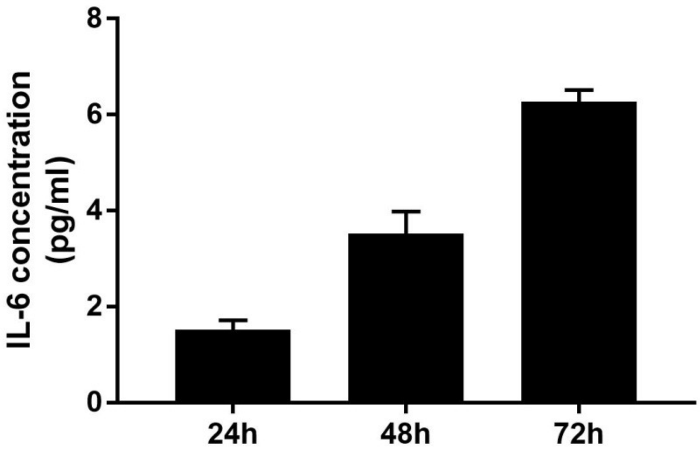

Supplementary Figure S2

Supplementary Figure S2: IL-6 production in HRMEC. HRMEC were transferred to starvation medium (MV2 medium + 0.5% FPS, but lacking all other growth factors) for 24h prior to the experiment and IL-6 was determined after 24, 48, and 72h by Luminex. Little IL-6 was detected but it accumulates with time.
